# Supplementary material for: Exploring factors affecting the facilitation of nursing students to learn paediatric pain management in Rwanda: A descriptive qualitative study
Source: PLoS One. 2022 Feb 16;17(2):e0263609. doi: 10.1371/journal.pone.0263609 (PMC8849445; doi:10.1371/journal.pone.0263609)
Supplement: S1 File — (DOCX) [file pone.0263609.s001.docx]

**Semi-structured interview guide for Nurse Educators and Preceptors (English)**

**Demographic questions**

Firstly, would you please provide some information about yourself.

1. Gender: Female

Male

1. Age group:

20-30 years

31-40 years

41 years and above

1. Highest obtained qualification: ----------------------------
2. Name of the institution where you work -----------------------
3. How many years of experience do you have with facilitating nursing students’ learning? -----
4. How long have you been facilitating nursing students’ learning about paediatric pain management? ---------------------

**Questions on facilitation of students learning paediatric pain management**

1. What training if any, have you received on paediatric pain management?
2. What personal or professional experiences will be required by you to facilitate competency acquisition for paediatric pain management by nursing students?
3. In your view, how capable are nurse educators/ preceptors in your settings in facilitating students learning paediatric pain management?
4. What do you consider to be an effective facilitation of competency acquisition for paediatric pain management by nursing students?
5. What will you suggest in order to enhance the capacity of nurse educators and preceptors to facilitate competency acquisition for paediatric pain management by nursing students?
6. What resources must be put in place for nurse educators’ and preceptors’ capacity enhancement to facilitate nursing students learning paediatric pain management?
7. What challenges or concerns have you experienced during the facilitation of students’ learning paediatric pain management?
8. In your view, what should be done to address those challenges to support students learning paediatric pain management?

Would you want to add anything?

**Inyoborakiganiro -Ibibazo byagenewe abarimu b´abaforomo**

**n´ababamenyereza umwuga ku bitaro**

**Ibibazo by’umwirondoro bibazwa uwitabiriye ubushakashatsi mu ntangiriro z’ikiganiro.**

Mwatwibwira.

1. Igitsina: Gore

Gabo

1. Ikigero cy’imyaka y’amavuko, hagati ya:

20-30

31-40

41 no kuzamura

1. Icyiciro cyo hejuru cy’amashuri wize…………..
2. Izina ry’ikigo mukorera -----------------------
3. Mufite uburambe bungana gute mu gufasha abanyeshuli babaforomo kubona ubumenyi n’ubumenyingiro? ------
4. Mufite uburambe bungana gute mu gufasha abanyeshuli kubona ubumenyingiro ku kuwita ku bana bafite ububabare? ---------------------

**Ibibazo bigize ikiganiro:**

1. Ni ayahe mahugurwa mwaba mwarabonye ajyanye no ku kwita ku bana bafite ububabare? ------------------
2. Ni ibiki mu kazi cyangwa mubuzima bwa buri munsi mwavuga ko bigira uruhare k’ubushobozi bwanyu bwo gufasha abanyeshuli b’abaforomo kubona ubumenyingiro ku kwita kubana bafite ububabare?
3. Uko mubona, abarimu b’abaforomo na abamenyereza mwuga aho mukora baba bafite ubushobozi buganabute bwo gufasha abanyeshuli b’abaforomo kubona ubumenyingiro ku kwita kubana bafite ububabare?
4. Ni iki mwumva cyaba kigize ubufasha bwuzuye ku banyeshuli b’abaforomo kubona ubumenyingiro ku kwita kubana bafite ububabare?
5. Mubona ari iki cyakorwa ngo hongerwe ubushobozi bwo abarimu b’abaforomo na abamenyereza mwuga ku gufasha abanyeshuli b’abaforomo kubona ubumenyingiro ku kwita kubana bafite ububabare?
6. Ni ibihe byangombwa nkenerwa kugira ngo hongerwe ubushobozi bw’abarimu ba baforomo n’abamenyereza umwuga mubijyanye ngo bafashe abanyeshuri ba baforomo kubona ubumenyingiro ku kwita ku bana bafite ububabare?
7. Ni izihe mbogamizi cyangwa ibibazo mwahuye nabyo mugihe cyo gufasha abanyeshuli kubona ubumenyingiro ku kwita ku bana bafite ububabare?
8. Mumva ari iki cyakorwa mukuvanaho izo mbogamizi muguteza imbere ubumenyi n’ubumenyingiro bw’abanyeshuli babaforomo ku kwita ku bana bafite ububabare?

Hari icyo mwifuza kongeraho?
