# Supplementary material for: Exploring factors affecting the facilitation of nursing students to learn paediatric pain management in Rwanda: A descriptive qualitative study
Source: PLoS One. 2022 Feb 16;17(2):e0263609. doi: 10.1371/journal.pone.0263609 (PMC8849445; doi:10.1371/journal.pone.0263609)
Supplement: S2 File — (DOCX) [file pone.0263609.s002.docx]

**Focus Group Discussions with nursing students**

**Demographic questions**

First, I would like to know some information about you:

1.Gender

Female

Male

##### 2. Age range: 19 – 24 years

##### 25-30yrs

31yrs and above

3. Nursing program:

Advanced Diploma (A1) year 3

Bachelor (BSN) year 4

3.Name of the institution where you study (public/ private)-----------------------

**Questions on facilitation of students learning paediatric pain management:**

This discussion will be mainly oriented on how you, as nursing students, have been facilitated to learn about paediatric pain management.

1. First of all, can you tell me what and how you have learnt about paediatric pain management?
2. How would you appraise the facilitation done by nurse educators and preceptors for your learning about paediatric pain management?
3. Can you please explain any difficulties you have experienced during students’ facilitation, either by educators at nursing school or preceptors in clinical setting, for competency acquisition for paediatric pain management?
4. What would you want to be changed, and in what way, about students’ facilitation by nurse educators and preceptors to acquire competence for paediatric pain management? (probing question).

You are welcome to provide any suggestion or comments.

**Inyoborakiganiro-Ibibazo bizifashishwa mu ikiganiro mvugo**

**n’itsinda ry’abanyeshuri biga igiforomo**

**Umwirondoro**

Mbere na mbere, Ndifuza kumenya amwe mu makuru abarekeye:

1. Igitsina: Gore

Gabo

2. Ikigero c’imyaka y’amavuko, hagati ya:

##### 19 – 24

##### 25 - 30

31 no kuzamura

3. Porogramu wigamo:

Umwaka wa 3 w’icyiciro cya mbere ya kaminuza (A1 year 3)

Umwaka wa 4 w’icyiciro cya kabiri cya kaminuza (BSN year 4)

4. Izina ry’ikigo wigamo -----------------------

**Ibibazo bijyanye n’uburyo mufashwa kwiga kwita ku bana bafite ububabare**

Iki kiganiro kiribanda ku buryo mwe nka abanyeshuri mwagiye mufashwa mubujyanye no kwiga kwita kubana bafite ububabare.

1. Mbere na mbere, mushobora kumbwira ni ibiki mwize bijyanye no kwita kubana bafite ububabare, mwabyize mute?
2. Mubona mute ubufasha abanyeshuri bahabwa n’abarimu ba baforomo n’abamenyereza mwuga mubijyanye no kwiga kwita ku bana bafite ububabare? Mushora kubivugaho mu buryo burambuye.
3. Mushobora gu sobanurira ingorane mwaba mwarahuye nazo haba mw’ishuri cg mugihe cyo kwiga ubumenyingiro mu bitaro mugihe abarimu n’abamenyereza mwuga babafashaga kubona ubumenyi n’ubumenyingiro ku kwita ku bana bafite ububabare?
4. Mubona ari iki cyakorwa kugirango abanyeshuri biga igiforomo barusheho bahabwa ubufasha bunoze bahabwa n’abarimu ba baforomo n’abamenyereza mwuga kigira ngo babone ubumenyingiro ku kwita ku bana bafite ububabare?

Murakose. Hari izindi nyunganizi cyangwa ibitekerezo mwabitanga.
