# Supplementary material for: Exploring factors affecting the facilitation of nursing students to learn paediatric pain management in Rwanda: A descriptive qualitative study
Source: PLoS One. 2022 Feb 16;17(2):e0263609. doi: 10.1371/journal.pone.0263609 (PMC8849445; doi:10.1371/journal.pone.0263609)
Supplement: S1 Table — (DOCX) [file pone.0263609.s003.docx]

**Participants demographic characteristics**

| **Participants in individual interviews** | **N = 14** |
| --- | --- |
| Occupation |  |
| Nurse educator | 6 |
| Nurse preceptor | 8 |
| Gender |  |
| Male | 6 |
| Female | 8 |
| Age (Mean: 34.5 years) |  |
| 20-30 years | 5 |
| 31-40 years | 7 |
| 41 years and above | 2 |
| Education level |  |
| Advanced Diploma in Nursing (3years) | 3 |
| Baccalaureate degree in Nursing | 8 |
| Master's in Nursing | 3 |
| Employment Setting |  |
| Academic Institution | 6 |
| Teaching hospital | 3 |
| Referral hospital | 3 |
| District Hospital | 2 |
| Experience in facilitating  students' learning: overall average in years | 4.7 years |
| Experience in facilitating students’ learning in paediatric pain  management : overall average in years | 2.8 years |
| Formal education in paediatric pain management after initial educational preparation |  |
| yes | 5 |
| no | 9 |
| **Participants in FGDs** | **N=19** |
| Occupation |  |
| Nursing students | **19** |
| Gender |  |
| Male | 13 |
| Female | 6 |
| Age (mean age: 23.7years) |  |
| 19- 24 years | 11 |
| 25-30 years | 6 |
| 31 years and above | 2 |
| Participants according to nursing program they are enrolled in |  |
| Advanced Diploma | 9 |
| Baccalaureate degree | 10 |
| Participants by type of academic institution they attend |  |
| Public | 10 |
| Private | 9 |
