# Supplementary material for: Exploring factors affecting the facilitation of nursing students to learn paediatric pain management in Rwanda: A descriptive qualitative study
Source: PLoS One. 2022 Feb 16;17(2):e0263609. doi: 10.1371/journal.pone.0263609 (PMC8849445; doi:10.1371/journal.pone.0263609)
Supplement: Attachment — Submitted filename: Response to Reviewers.docx [file pone.0263609.s004.docx]

**Rebuttal letter**

Dear Editor,

Thank you for taking your time to review our manuscript and for giving us the opportunity to respond to your comments and those of the reviewers. I wish to express my appreciation to the reviewers for their constructive feedback which I hope helped improving the improvement of the manuscript as I worked to respond to all the comments provided.

This table depicts how all the issues raised were addressed and the page and line numbers correspond to the manuscript with track changes.

| **N** | **Section with comments** | **Reviewer’s comments** | **How the comments were addressed** | **Section, page/line where comments are addressed** |
| --- | --- | --- | --- | --- |
| **Academic Editor** | | | | |
| **1.** |  | Please ensure that your manuscript meets PLOS ONE's style requirements, including those for file naming. | Thank you. The manuscript was formatted to meet PLOS ONE’ style requirements | Whole document |
| **2.** |  | We note that the grant information you provided in the ‘Funding Information’ and ‘Financial Disclosure’ sections do not match.  When you resubmit, please ensure that you provide the correct grant numbers for the awards you received for your study in the ‘Funding Information’ section. | Thank you for this comment. The project that support part of the expenses related to the study data collection, the Training, Support and Access Model (TSAM) for Maternal Newborn and Child Health (MNCH) in Rwanda was a project from the Western University and was funded by Global Affairs Canada.  The grant number is provided in the section on ‘Funding Information’ | In the submission system |
| **3.** |  | In your Data Availability statement, you have not specified where the minimal data set underlying the results described in your manuscript can be found.  Upon re-submitting your revised manuscript, please upload your study’s minimal underlying data set as either Supporting Information files or to a stable, public repository and include the relevant URLs, DOIs, or accession numbers within your revised cover letter. | Thank you. All relevant data are within this manuscript and its Supporting information files. |  |
| **4.** |  | We note that you have indicated that data from this study are available upon request.  In your revised cover letter, please address the following prompts:  a) If there are ethical or legal restrictions on sharing a de-identified data set, please explain them in detail (e.g., data contain potentially sensitive information, data are owned by a third-party organization, etc.) and who has imposed them (e.g., an ethics committee). Please also provide contact information for a data access committee, ethics committee, or other institutional body to which data requests may be sent.  b) If there are no restrictions, please upload the minimal anonymized data set necessary to replicate your study findings as either Supporting Information files or to a stable, public repository and provide us with the relevant URLs, DOIs, or accession numbers. | Thank you. This was addressed and a part from all data are in this paper, supporting information files are uploaded.  Captions for the Supporting Information files were included at the end of the manuscript and in-text citations done accordingly. | Page 7, line 175, line 180; page 8 line 200; page 10 line 268  Page 27, lines 735-739 |
| **4.** |  | Your ethics statement should only appear in the Methods section of your manuscript. If your ethics statement is written in any section besides the Methods, please delete it from any other section. | Thank you. It was noted, the ethics statement is captured under the Methods section and not elsewhere | Pages 8-9 from line 229 -242 |
| **Reviewer #1: PONE-D-21-20434** | | | | |
| **1.** | Keywords | Should they be presented in alphabetical order? | Thank you for the suggestion. The Keywords are presented following alphabetic order. | In the submission system. |
| **2.** | **INTRODUCTION** | Line 39- 41: A reference would support this important statement which is a rational for the entire study. | Thank you. Two references were added to support the statement. | Page 3 from line 45- 48 |
| **3.** |  | Line 68 (+ abstract): could “the effective” be omitted in the aim? …”explored factors affecting the effective facilitation of nursing students for learning about PPM as perceived by academics, clinical nurse preceptors, and nursing students in Rwanda.” | Thank you for the suggestion. The word “ effective” was removed in the aim in the abstract and in the introduction | In the abstract line 9 and on page 4, line 75 |
| **4.** | **METHODS** | Line 76-77: Is this sentence “The study authors utilised this approach to explore the factors influencing the facilitation of nursing students’ learning about PPM” needed or just a way of rephrasing the aim? | Yes it was a way of rephrasing the aim of the study. The sentence was removed. | Page 5, line 103 |
| **5.** |  | The choice of qualitative approach was unclear to me when I first read the manuscript. Reference 15; Neergaard (2009) Qualitative description-the poor cousin of health research? as well as Qualitative Descriptive (QD) as an approach was unknown to me and I applogize if I that might interfere with some of my comments. However I have now read the reference and tried my best to understand the manuscript in relation to this. Further down in the methods authors claim that they have used a thematic analysis (TA). Neergaard et al. describe the possibility to within QD use the steps of content analysis within QD. Is it based on this information the authors of this manuscript have decided to include also TA? If so, the manuscript would benefit from a description of QD and a rational for why these two approach was chosen. Other parts of the method section should also benefit from adjustments in relation to QD. For example; have the authors really performed in-depth interviews and if so why when Neergaard promote semi-structured interviews. Is it really narratives that has been collected or rather experiences? | Thank you for the comment. A statement was added with supporting references to explain why qualitative descriptive approach was used.  Neergaard stated that content analysis is the most common in qualitative descriptive studies, but thematic analysis (TA) has also been used in many other research studies using qualitative descriptive approach. TA was used in this study as well. | Pages 4 and 5, from lines 79-98 |
| **6.** |  | Line 79: Authors state that the sites were chosen purposively and by the description it seems as if authors were choosing purposively to reach a variation in included sites. This could be clarified. | Thank you. The statement was refined for more clarification. It reads as follows:  *To reach a variety of study sites, these were chosen purposively taking into consideration whether they are public or private institutions, if they are located in a rural or urban area, and for teaching institutions, if an advanced diploma and/or bachelor degree program of nursing is offered.* | Page 5 from line 105-108 |
| **7.** |  | Line 104: The statement that data was saturated after 6+8 informants is quite specific and the sentence would be strengthened by some further explanation on the matter. In what regard was the data saturated? Who decided? Is it in line with Neergaard et al./ QD to reach saturation? | Thank you for the comment. The statement was further explained and supported by additional references. | Page 6, lines 143- 145 |
| **8.** |  | Line 124: It is unclear to me what the reference is supporting? Total number of participants? The sentence is not telling us anything about focus group size. Furthermore somewhere in the method and/or discussion the choice of using both focus group and individual interviews should be described or reflected on. | Thank you. The sentence was rephrased as follows:  *The sample size of the FGDs was deemed sufficient in this study considering what was stated in the literature [26].*  Also, an explanation of why both focus group and individual interviews was provided. | Page 7, lines 161 – 163  Page 5, lines 98-103 |
| **9.** |  | Line 130: Can an interview be both semi structured and in-depth as previously stated? Could the authors clarify the content of the interview guide. It is stated in the manuscript, If I have understood it correctly, that this was built on findings from previous studies. In what way or to what extent?? | Thank you. A summary of the content of interview guides for both IDIs and FGDs was included in the methods.  Both interview guides were uploaded as Supporting files (S1 and S2) | Page 7, from line 177-180 and page 8 lines 195 -200  Page 7, line 180 and page 8, line 200 |
| **10.** |  | Line 139-140; Could the information be more specific than generally 30-60 minutes? | Thank you for the suggestion. This was addressed | Page 8, lines 193- 194 |
| **11.** |  | Line 149-151: With whom were they provided this opportunity, when and why? | The sentence was rephrased as follows:  *Before ending of each interview, the primary investigator who conducted the interviews and facilitated the FGDs at the different times provided to the participants the opportunity to discuss any additional perspectives related to the facilitation of students’ competency acquisition for PPM that were not shared during IDI or FGD.* | Page 8, lines 206- 209 |
| **12.** |  | Line 154: The use of codes and pseudonyms can only be considered anonymous if there is no code-key or anything otherwise consider changing to “confidentiality”. | This was fixed. | Page 8, line 212 |
| **13.** |  | Line 175: It is unclear to me what authors mean by “the codes were combined to form larger meaningful segments using the deductive analytic technique that enabled the conceptualization of themes”. Was a deductive analysis really performed and if so based on what theory or model? It does not correspond to other statements in the method section. | Thank you for the comment. The statement was revised, and hopefully it is clearer. It reads:  *The coding process was interactive using the line-by-line technique to create initial codes. Then initial codes were combined into larger meaningful segment codes from which the themes emerged. The use of inductive coding approach allowed the development of unanticipated themes from the raw data [32].* | From page 9, lines 240 - 243 |
| **14.** | **RESULTS** | Line: 218: Is this part of the quote adequate for the theme? ”Such a student leaves the learning environment with gained knowledge. So, students have to be well focused when they come into clinical placement with daily objectives that they have to achieve.”? Or could this last part be removed? | Thank you for the suggestion. The last part of quote was omitted | Page 11, line 293 |
| **15.** |  | Line 235: Is it clear who is the facilitator in this case? | It was explained in the following sentence. | Page 12, lines 312-313 |
| **16.** |  | Line 288: Could this Theme be rephrased? The content is interesting but the title “Nurses' restrictive scope of practice…” is unclear to me. | Thank you for the comment. The theme was changed to “Nurses’ limited autonomy for decision-making regarding PPM practices” | In the abstract lines 19-20; on page 10, lines 271-272 and page 14, lines 365-366 |
| **17.** |  | Line 290-292: Is the sentence “To some extent nurses are still perceived as physicians’ assistants with limited authority to choose paediatric pain relief practices.” derived from data or is it a statement from the authors? Possibly rephrase the sentence for clarification. (If from the authors remove) | Thank you for the suggestion. This was addressed to reflect that it was derived from participants | Page 14, lines 368-369 |
| **18.** |  | Line 306: Could this Theme be rephrased to be more specific The resources discussed is human and material resources. | This was done, thank you. | In the abstract lines 19-20,  Page 10 lines 272, and on page 15, line 391 |
| **19.** | **DISCUSSION** | Wordy but overall relevant and nice discussion  Line 400-4001: Should the following be included in the results in order to be discussed? “As stated by [31] “personality traits can help in the aligning of students” and clinical instructors, as well as can cause rifts in the teaching relationship”(p.424) | Thank you.  It is included. It states : *A nurse educator reiterated this by maintaining that personality traits of facilitators contribute in one way or another to how s/he would facilitate students to acquire knowledge and skills.* | Page 12, lines 320-321 |
| **20.** | **STRENGTHS AND LIMITATIONS** | Line 496-499: I would not consider it a limitation for the study as such that it “represent only personal views of the study participants” as this was the purpose of the study. | The statement on limitation was rephrased | Page 23, line 579-582 |
| **21.** | **REFERENCES** | Most references are at least 3 years old. Would it be possible to supplement with more resent research findings? | Thank you for the suggestion. More recent references were added (around five of them) | Page 24, lines 623 and 651, page 25, lines 653, 657, 666, 679 |
| **Reviewer #2** | | | | |
|  | Thank you to the second reviewer for the positive feedback. | | There was no comment that required to be responded to. |  |
